# Mental health impact of autism on families of children with intellectual and developmental disabilities of genetic origin

**DOI:** 10.1002/jcv2.12128

**Published:** 2023-01-13

**Authors:** Jeanne Wolstencroft, Ramya Srinivasan, Jeremy Hall, Marianne B. M. van den Bree, Michael J. Owen, IMAGINE Consortium, F. Lucy Raymond, David Skuse

**Affiliations:** ^1^ UCL NIHR BRC Great Ormond Street Institute of Child Health University College London London UK; ^2^ UCL Division of Psychiatry University College London London UK; ^3^ Medical Research Council Centre for Neuropsychiatric Genetics and Genomics Division of Psychological Medicine and Clinical Neurosciences Cardiff University Cardiff UK; ^4^ Neuroscience and Mental Health Research Institute Cardiff University Cardiff UK; ^5^ School of Clinical Medicine University of Cambridge Cambridge UK; ^6^ Cambridge University Hospitals NHS Foundation Trust Cambridge Biomedical Campus Cambridge UK; ^7^ NIHR Bioresource Cambridge Biomedical Campus Cambridge UK

**Keywords:** autism, genetic disorder, intellectual disability

## Abstract

**Background:**

Many children with an intellectual or developmental disability (IDD) have associated autism spectrum disorders (ASD), as well as an increased risk of mental health difficulties. In a cohort with IDD of genetic aetiology, we tested the hypothesis that excess risk attached to those with ASD + IDD, in terms of both children's mental health and parental psychological distress.

**Methods:**

Participants with a copy number variant or single nucleotide variant (5–19 years) were recruited via UK National Health Service. 1904 caregivers competed an online assessment of child mental health and reported on their own psychological wellbeing. We used regression to examine the association between IDD with and without co‐occurring ASD, and co‐occurring mental health difficulties, as well as with parental psychological distress. We adjusted for children's sex, developmental level, physical health, and socio‐economic deprivation.

**Results:**

Of the 1904 participants with IDD, 701 (36.8%) had co‐occurring ASD. Children with both IDD and ASD were at higher risk of associated disorders than those with IDD alone (ADHD: OR = 1.84, 95% confidence interval [CI] 1.46–2.32, *p* < 0.0001; emotional disorders: OR = 1.85, 95%CI 1.36–2.5, *p* < 0.0001; disruptive behaviour disorders: OR = 1.79, 95%CI 1.36–2.37, *p* < 0.0001). The severity of associated symptoms was also greater in those with ASD (hyperactivity: *B* = 0.25, 95%CI 0.07–0.34, *p* = 0.006; emotional difficulties: *B* = 0.91, 95%CI 0.67 to 1.14, *p* < 0.0001; conduct problems: *B* = 0.25, 95%CI 0.05 to 0.46, *p* = 0.013). Parents of children with IDD and ASD also reported greater psychological distress than those with IDD alone (*β* = 0.1, 95% CI 0.85 to 2.21, *p* < 0.0001). Specifically, in those with ASD, symptoms of hyperactivity (*β* = 0.13, 95% CI 0.29–0.63, *p* < 0.0001), emotional difficulties (*β* = 0.15, 95% CI 0.26–0.51, *p* < 0.0001) and conduct difficulties (*β* = 0.07, 95% CI 0.07–0.37, *p* < 0.004) all significantly contributed to parental psychological distress.

**Conclusions:**

Among children with IDD of genetic aetiology, one third have co‐occurring ASD. Not only do those with co‐occurring ASD present with a wider range of associated mental health disorders and more severe mental health difficulties than those with IDD alone, but their parents also experience more psychological distress. Our findings suggest that the additional mental health and behavioural symptoms in those with ASD contributed to the degree of parental psychological distress.

**Key points**

- Children with an intellectual or developmental disabilities (IDDs) with or without associated Autism Spectrum Disorder (ASD) are at increased risk of mental health and behavioural difficulties and their parents are reported to have higher levels of psychological distress compared to the parents of neurotypical children.
- However, little is known about these issues in children whose intellectual or developmental disability (IDD) has a genetic cause. With rapid increases in genetic testing, we urgently need to know more about mental health difficulties and parental psychological distress in children of IDD of genetic cause to develop and provide more individualised support.
- We found that children with both IDD and co‐occurring ASD were two times more likely to meet criteria for additional disorders such as Attention Deficit Hyperactivity Disorder, emotional disorders and disruptive behaviour disorders compared to those with IDD alone.
- Parental psychological distress in families whose child has both ASD and IDD appears to be magnified by the presence of these additional mental health problems.
- The presence of ASD in addition to IDD in children with IDD of genetic cause should alert clinicians to the possibility of elevated mental health problems in children, and the need for a comprehensive assessment of psychiatric disorders; and they may require additional behavioural and mental health support.

## INTRODUCTION

Intellectual and developmental disabilities (IDDs) are characterised by significant limitations in cognitive and adaptive skills (American Association on Intellectual and Developmental Disabilities., 2010). Intellectual or developmental disability frequently co‐occurs with autism spectrum disorder (ASD) (Baio et al., 2018; Fombonne, 2005), which is characterised by deficits in reciprocal social interaction and repetitive and/or stereotyped behaviours (American Psychiatric Association & DSM‐5 Task Force, 2013). Estimates of IDD and ASD co‐occurrence vary substantially; between 4% and 40% of children with IDD may have co‐occurring ASD (Bryson et al., 2008; Emerson, 2003; Emerson & Hatton, 2007; Koskentausta et al., 2002; Lakhan, 2013; la Malfa, Lassi, Bertelli, Salvini, & Placidi, 2004). The proportion of children with ASD who have IDD is uncertain. Historically, studies tended to ascertain those at risk based on early developmental delay (Bertrand et al., 2001; Chakrabarti & Fombonne, 2001, 2005; Fombonne, 2005, 2009; Maenner et al., 2021) and most recent research has focussed on ASD in association with normal‐range Intelligence Quotient (Russell et al., 2019).

Both IDD and ASD are largely genetic in aetiology. An identified genomic cause can be found in up to 60% of children with an IDD (Gilissen et al., 2014) and between 10% and 30% of children with ASD (Buxbaum, 2009; Ronemus et al., 2014; Sanders et al., 2015). Genetic testing is now part of routine clinical care when IDD is suspected in children in the UK. There is some evidence that children with identified genomic disorders have a higher risk of neurodevelopmental disorders in general (Chawner et al., 2021; Chawner et al., 2019) although the prevalence rates of ASD could vary between disorders considerably (Richards, Jones, et al., 2015). Our understanding of how best to support children with rare genetic disorders once they have been diagnosed is lagging behind testing capacity, but the Intellectual Disability and Mental Health: Assessing the Genomic Impact on Neurodevelopment (IMAGINE) consortium aimed to bridge that knowledge gap by collecting data on the development and wellbeing of over 1900 children ascertained for IDD of known genetic aetiology.

Both children with an IDD or ASD are at increased risk of internalising (emotional problems, anxiety disorders) and externalising problems (conduct problems, impulsivity, aggression) (Bauminger et al., 2010; Dekker et al., 2002; Dykens, 2000), which can be a consistent concern from childhood through adolescence (Einfeld et al., 1999; Tonge & Einfeld, 2000; Totsika & Hastings, 2009). Furthermore, IDD and ASD have been shown to be independent risk factors for mental health difficulties in children (Totsika, Hastings, Emerson, Lancaster, & Berridge, 2011). Whilst there is a growing interest in the mental health of children with ASD, the study of children with co‐occurring IDD and ASD has been neglected (Happé & Frith, 2020; Russell et al., 2019). To date, the impact of co‐occurring ASD and IDD on mental health problems has only been examined in subsets derived from national cohorts of the general population, resulting in modest numbers of children with ASD and both ASD and IDD (Totsika, Hastings, Emerson, Berridge, & Lancaster, 2011; Totsika, Hastings, Emerson, Lancaster, et al., 2011). Consequently, the mental health risks associated with co‐occurring IDD and ASD are poorly understood. In this study, we aimed to evaluate those risks in a large nationally representative cohort of children who were recruited based on an NHS‐diagnosed genetic aetiology for their developmental delay. We wanted to examine mental health using symptom based continuous measures, as well as diagnostic categorical measures in order to take into account difficulties that have a substantial functional impact, without meeting diagnostic criteria. On the basis of previous research (Wolstencroft et al., 2022) we know over one‐third of our cohort had an ASD, and we aimed to test the hypothesis that children with co‐occurring ASD and IDD of genetic aetiology would have more mental health problems, than those with IDD alone. We hypothesized that children with co‐occurring ASD and IDD of genetic aetiology would have more mental health problems, than those with IDD alone.

Not only do children with IDD and those with ASD have an excess risk of associated mental health problems but their parents also have elevated psychopathology compared to the parents of typically developing children (Baker‐Ericzén et al., 2005; Blacher & McIntyre, 2006; Emerson, 2003; Feldman et al., 2007; Gray et al., 2011; Marquis et al., 2019; Mugno et al., 2007; Olsson & Hwang, 2001; White & Hastings, 2004). The range and severity of associated externalising and internalising domains of symptomatology (e.g., hyperactivity, social skills, anxiety, depression, cognitive ability) associated with diagnoses of ASD and/or IDD vary substantially from individual to individual. We aimed to assess whether specific domains are associated with a greater risk parental psychological distress, in order to identify meaningful targets for intervention (Hayes & Watson, 2013).

Our objectives were threefold: first, to quantify the strength of the association between IDD (with and without occurring ASD) and mental health diagnoses; second, to quantify the strength of association between IDD (with and without ASD) and the severity of mental health symptoms measured dimensionally; third, to identify the most prominent behavioural difficulties that are associated with parental psychological distress, in those with IDD compared with participants with co‐occurring IDD and ASD.

## METHODS

### Recruitment

Two thousand seven hundred and seventy children aged 4–19 years with IDD of genetic origin consented to participate in IMAGINE and 2397 (86.5%) completed at least one assessment. The inclusion criteria were (i) a clinically documented developmental delay or intellectual disability, (ii) a pathogenic molecular genetic diagnosis documented from an accredited diagnostic laboratory (a copy number variant, CNV; or single nucleotide variant (SNV). Variant pathogenicity was classified according to American College of Medical Genetics guidelines (Richards, Aziz, et al., 2015), (iii) at least 4 years of age at recruitment, (iv) and an available caregiver or parent to report on the child's behaviour. Participants were recruited between 2014 and 2019 from UK regional genetics centres (76%) and self‐referrals (24%). Informed consent was obtained from parents or caregivers of all child participants aged under 16 years old, and an assent form was completed by the child where possible. Consultee forms were completed by the parents or caregivers of children over the age of 16 who lacked capacity to consent on their own behalf. All procedures were approved by London–Queen Square Research Ethics Committee (14/LO/1069).

Assessments were administered using the Developmental and Wellbeing Assessment (DAWBA). They were completed either online (80%), over the phone or in person based on the family's preferred mode of completion.

### Measures

Mental health and neurodevelopmental disability were assessed using both categorical diagnostic (DSM‐5) and continuous symptom‐based measures; to ensure that mental health difficulties that may have significant functional impact, but which do not meet diagnostic criteria were accounted for. We also used modules of the DAWBA which collected information on parental psychological distress, and child and family characteristics.

#### DSM‐5 diagnoses

The DAWBA survey was completed online by the parent or primary caregiver. The DAWBA online portal is organised into modules which collect information about the child's family environment, schooling, behavioural adjustment and psychopathology, as well as the wellbeing of the caregiver. The DAWBA has been used both in UK national and international surveys of child psychopathology (Emerson & Hatton, 2007; Ford et al., 2003; Green et al., 2004; Heiervang et al., 2008). This study followed the DAWBA rating procedures used in the UK national surveys of child psychopathology. Parental answers are reviewed by trained clinical raters, who assign DSM‐5 diagnoses. The reliability of DAWBA diagnoses has been consistently high in studies of inter‐rater reliability in population‐based studies (Aebi et al., 2012; Fleitlich‐Bilyk & Goodman, 2004; Ford et al., 2003; Heiervang et al., 2007; Office for National Statistics. Social and Vital Statistics Division et al., 2004) and in the IMAGINE cohort (*n* = 308; *k* = 0.83; Coscini et al., 2020). Clinical ratings have also been validated on a subsample of 29 participants, who were independently assessed for ASD using the Developmental Diagnostic Dimensional Interview‐Short Version (3Di‐sv). The 3Di‐sv is a 45‐min standardized parental interview for ASD assessment administered by trained clinical interviewers (Santosh et al., 2009; Skuse et al., 2004). The 3Di‐sv has excellent concurrent validity, discriminant validity, and criterion validity (Santosh et al., 2009; Skuse et al., 2004). The agreement between the clinical and the DAWBA and 3Di raters was high (*k* = 0.722; *p* < 0.0005); with excellent sensitivity (93.3%) and specificity (78.6%).

#### Mental health symptoms

The Strengths and Difficulties Questionnaire (SDQ) is a well‐validated screening questionnaire for children, including those with IDD (Goodman et al., 2010; Murray et al., 2021) (Cronbach alpha in this sample 0.8). The SDQ includes scales that measure emotional symptoms, conduct problems, hyperactivity/impulsivity and inattention difficulties, peer relationship problems and prosocial behaviour. The first four scales are combined to create a total difficulties score. High scores are indicative of greater levels of mental health difficulty and scores above the 90th percentile indicate a high probability of a diagnosable psychiatric disorder (≥17 by parent report; ≥18 by self‐report) (Goodman, 2001).

#### Parental psychological distress

The Everyday Feelings Questionnaire (EFQ) is a short 10‐item measure of psychological wellbeing and distress in adults. The EFQ is an internally consistent and externally valid measure of psychological well‐being and distress (Goodman & Goodman, 2009; Uher & Goodman, 2010) (Cronbach alpha in this sample 0.9). Parents and caregivers reported on themselves.

#### Child and family characteristics

The DAWBA's background section collects information on the child's schooling, physical health, and parental estimates of mental age. A developmental quotient was computed using the parent's estimate of their child's mental age as the numerator and the child's chronological age as the denominator (Baker et al., 2021; Emerson & Hatton, 2007). Developmental quotients range between 0 and 1, the latter indicating chronological age and mental age are equivalent. General physical health was estimated on a 5‐point Likert scale from very bad to very good.

#### Indices of multiple deprivation

Socio‐economic status was ascertained through postcode data collected at study enrolment using the Indices of multiple deprivation (IMD) (Office for National Statistics (ONS), 2019). Indices of multiple deprivation scores combine information from seven domains to produce a relative measure of deprivation. The domains include income, employment, education, health, crime, barriers to housing and services, and the living environment. Indices of multiple deprivation scores are ranked and organised into deciles; the first decile includes the most deprived postcodes, and the 10th decile includes the least deprived postcodes. Indices of multiple deprivation scores are available for England, Scotland, Wales, and Northern Ireland.

### Sample characteristics

Complete DAWBA data were available on 1904 participants. The mean age of participants was 9.15 years (SD = 3.75, range 4–19 years, Table 1). Following genetic testing, which included a microarray and/or exome sequencing, over two‐thirds of the overall sample were found to have one or more copy number variant (*n* = 1328; 69.7%), and the remainder had a pathogenic SNV (*n* = 487; 25.6%) or a sex chromosome aneuploidy (*n* = 89; 4.7%). Just over half of the overall sample was male (*n* = 1072; 56.3%). Most participants attended special school or a special unit in a mainstream school (*n* = 812; 42.6%) or mainstream school with some extra help (805; 42.3%). A small proportion of participants attended ordinary school without extra help (101; 5.3%) or didn't attend school (107; 4.1%). The school provision was not recorded for 79 (5.6%) participants. Three quarters of the families (75.8%; *n* = 1444) reported receiving help from a paediatric professional, but only 28.9% (*n* = 550) reported receiving help from a mental health professional. Parents of autistic children were more likely to report receiving help from paediatric (88.5% vs. 73.3%) and/or mental health professionals (37.2% vs. 24.2%) than those with IDD alone.

**TABLE 1:** Sample demographics

	All	ASD	No ASD	*p*‐value
	*n* = 1904	*N* = 701	*N* = 1203	
Participant characteristics				
Male, no. (%)	1072 (56.3)	450 (64.2)	622 (51.7)	<0.001
Age, mean (SD)	9.15 (3.75)	9.19 (3.9)	9.12 (3.67)	0.07
General health score, mean (SD)	1.13 (0.91)	1.07 (0.88)	1.24 (0.96)	<0.001
Developmental quotient, (mental age/chronological age), mean (SD)	0.55 (0.23)	0.54 (0.24)	0.55 (0.2)	0.43
IMD, no. (%)				0.02^a^
1–2 (most deprived)	359 (18.9)	145 (20.7)	214 (17.8)	
3–4	357 (18.7)	150 (21.4)	207 (17.2)	
5–6	353 (18.6)	118 (16.8)	232 (19.6)	
7–8	399 (20.9)	147 (21)	252 (20.9)	
9–10 (least deprived)	436 (22.9)	141 (20.1)	295 (24.5)	
DAWBA DSM‐5 diagnoses^b^				
Autism spectrum disorder, No. (%)	701 (36.8)	‐	‐	‐
Hyperactivity disorder, No. (%)	422 (22.2)	217 (31)	205 (17)	<0.001
Emotional disorders, No. (%)	198 (10.4)	105 (15)	93 (7.7)	<0.001
Generalised anxiety, No. (%)	126 (6.6)	72 (10.3)	54 (4.5)	<0.001
Separation anxiety, No. (%)	35 (1.8)	14 (2)	21 (1.7)	0.69
Specific phobia, No. (%)	49 (2.6)	23 (3.3)	26 (2.2)	0.14
Social phobia, No. (%)	3 (0.2)	1 (0.1)	2 (0.2)	0.90
Obsessive Compulsive Disorder, No. (%)	1 (0.1)	1 (0.1)	1 (0.1)	0.70
Major Depression, No. (%)	8 (0.4)	5 (0.7)	3 (0.2)	0.13
Conduct disorders, No. (%)	263 (13.8)	143 (20.4)	120 (10)	<0.001
ODD	248 (13)	137 (19.5)	111 (9.2)	<0.001
CD	24 (1.3)	12 (1.7)	12 (1)	0.18
SDQ subscale scores				
Emotional symptoms, mean (SD)	4.66 (2.82)	5.43 (2.76)	4.21 (2.75)	<0.001
Hyperactivity symptoms, mean (SD)	7.9 (2.1)	8.28 (1.89)	7.68 (2.13)	<0.001
Conduct problems, mean (SD)	3.44 (2.4)	3.94 (2.36)	3.15 (2.37)	<0.001
Parental distress				
EFQ, mean (SD)	16.93 (7.36)	18.04 (7.5)	16.28 (7.2)	<0.001

^a^ Two sample Kolmogorov–Smirnov test.

^b^ Chi squared test.

Overall, 36.8% (*n* = 701) of all participants met criteria for an ASD. The male‐female ratio in those meeting criteria for an ASD in addition to IDD was 1.8 to 1, whereas the sex ratio was equal in those with IDD alone (Table 1). The degree of developmental impairment, as indexed by the developmental quotient was comparable in both groups (Table 1). Physical health was rated as better in those that met criteria for a co‐occurring ASD diagnosis compared to those with IDD alone (Table 1). The socio‐economic status of participants, as indexed by the IMD score approximated a normal distribution (Table 1).

The prevalence of mental health and neurodevelopmental disorders was high in the sample overall. Yet, there was a higher prevalence of co‐occurring mental health difficulties and diagnoses, in those with ASD and IDD compared to the those with IDD alone. Parent psychological distress was also higher in parents of children with co‐occurring ASD and IDD compared to those with IDD alone (Table 1).

### Statistical analysis

All analyses were conducted in SPSS version 25 (IBM Corp, 2017). Descriptive statistics were used to describe the sample (means, standard deviations, frequencies, and proportions). Independent group statistics were used to compare the mental health profiles of children with and with an ASD on demographic variables and study outcome measures. Three sets of regression analyses were conducted to investigate the unique contribution of ASD in addition to IDD on child mental health, behaviour, and parental distress.

#### Analysis 1

In this set of analyses, we examined the contrast between children with IDD and those with an additional ASD, using binary logistic regression models and DSM‐5 diagnoses from the DAWBA as the dependent variable. Diagnoses included attention deficit hyperactivity disorder [ADHD]), emotional disorders, disruptive behaviour disorders). We first ran univariable binary logistic analyses and subsequently multivariable models that adjusted for IMD, sex, developmental level, physical disability, as well as other DSM‐5 diagnoses as covariates. We ran supplementary analyses using the individual DSM‐5 DAWBA diagnoses for emotional disorders and disruptive behaviour disorders (e.g. Generalised anxiety disorder [GAD], separation anxiety, specific phobia, obsessive compulsive disorder [OCD], major depression, disruptive behaviour disorders, oppositional defiant disorder [ODD], or conduct disorder [CD]).

#### Analysis 2

We then examined the association between IDD with and without co‐occurring ASD, with the dependent variable being mental health symptoms measured dimensionally, as indexed by the SDQ. First we ran a multiple linear regression with the SDQ total score as the dependent variable, where we included IMD, developmental level and physical disability as co‐variates. Secondly, we conducted a series of hierarchical multiple regression models with SDQ hyperactivity symptoms, SDQ emotional symptoms and SDQ conduct problems as the dependent variable. For each dependent variable we first entered IMD, developmental level and physical disability as co‐variates. We then included the other SDQ subscales as co‐variates.

#### Analysis 3

In this series of analyses, we examined the association between IDD with and without co‐occurring ASD and parental psychological distress as measured by the EFQ. In a hierarchical multiple regression model, we took the EFQ total score (measuring parental psychological distress) as the dependent variable. We first entered IMD, developmental level and physical disability, then added the SDQ hyperactivity, emotional and conduct subscales as a co‐variates. Standardised beta coefficients for SDQ mental health symptom domains are reported, to aid the interpretation of the importance of the contribution of each SDQ subscale to parental psychological distress.

#### Additional sensitivity analyses

We conducted sensitivity analyses to assess for differences in participants that completed questionnaires online and over the phone. Each analysis was repeated with the online completion participants.

## RESULTS

### Analysis 1: The association between intellectual or developmental disability with and without co‐occurring autism spectrum disorders and Developmental and Wellbeing Assessment DSM‐5 diagnoses

A series of binary logistic regression models investigated the association between IDD with and without co‐occurring ASD, and individual mental health diagnoses from the DAWBA DSM‐5 (ADHD, emotional disorders or disruptive behaviour disorders), using binary logistic regression models.

In Model 1 we found evidence that children with IDD and co‐occurring ASD were more likely to meet criteria for ADHD (OR 2.18, 95% confidence interval [CI] 1.75–2.72, *p* < 0.0001), for emotional disorders (OR 2.1, 95%CI 1.56–2.83, *p* < 0.0001), and for disruptive behaviour disorders (2.31, 95%CI 1.78–3.01, *p* < 0.0001).

The evidence for this association remained significant in Model 2 after adjustment for covariates relating to the child and their family environment (sex, developmental level, physical health, deprivation); ADHD (OR 2.08, 95%CI 1.66–2.6, *p* < 0.0001); emotional disorders OR (2.03 95%CI 1.5–2.74, *p* < 0.0001); disruptive behaviour disorders (OR 2.18, 95%CI 1.66–2.85, *p* < 0.0001), and for Model 3 which included additional co‐occurring disorders (ADHD OR 1.84, 95%CI 1.46–2.32, *p* < 0.0001; emotional disorders OR 1.85, 95%CI 1.36–2.5, *p* < 0.0001; disruptive behaviour disorders OR = 1.79, 95%CI 1.36–2.37, *p* < 0.0001; Table 2).

**TABLE 2:** Analysis 1: Odds ratios for child mental health diagnosis according to presence of intellectual or developmental disability (IDD) with or without co‐occurring autism spectrum disorders (ASD) using binary logistic regression models (*n* = 1904)

DAWBA Child DSM‐5 diagnosis (0 no ASD/1 ASD)	Model 1		Model 2		Model 3	
	OR (95% CI)	*p*‐value	OR (95% CI)	*p*‐value	OR (95% CI)	*p*‐value
Attention deficit hyperactivity disorder	2.18 (1.75, 2.72)	<0.0001	2.08 (1.66, 2.61)	<0.0001	1.84 (1.46, 2.32)	<0.0001
Emotional disorders	2.10 (1.56, 2.83)	<0.0001	2.03 (1.50, 2.74)	<0.0001	1.85 (1.36, 2.5)	<0.0001
Disruptive behaviour disorders	2.31 (1.78, 3.01)	<0.0001	2.18 (1.66, 2.85)	<0.0001	1.79 (1.36, 2.37)	<0.0001

*Note*: **Model 1** – univariable associations between IDD with or without co‐occurring ASD and DSM‐5 diagnosis. **Model 2** – Model 1 including confounding variables – child sex, child developmental level, deprivation (IMD), child physical disability. **Model 3** – Model 2 including co‐occurring DSM‐5 diagnoses as confounding variables.

Abbreviation: CI, confidence interval.

Additional analyses presented in the supplementary materials were conducted with individual DAWBA DSM‐5 diagnoses including generalised anxiety, specific phobia, social phobia, separation anxiety, OCD, depression, ODD and CD (Table S2). In these analyses we found evidence that children with IDD and co‐occurring ASD were more likely to meet criteria for GAD (OR 2.15, 95%CI 1.46–3.16, *p* < 0.001) and for ODD (OR 1.90 95%CI 1.44–2.56, *p* < 0.001; Table S2) in fully adjusted models.

### Analysis 2: The association between intellectual or developmental disability with and without co‐occurring autism spectrum disorders and mental health symptoms

A series of hierarchical multiple regression models were conducted to investigate the associations between IDD with and without co‐occurring ASD, and mental health difficulties as indexed by the SDQ subscales. Model 1 adjusted for deprivation, developmental level, sex, physical disability, and Model 2 for the other SDQ subscales.

In Model 1 there was evidence that ASD in addition to IDD significantly predicted SDQ total score (*B* = 3.7, 95%CI 3.18–4.3, *p* < 0.0001), hyperactivity symptoms (*B* = 0.5, 95%CI 0.32–0.7, *p* < 0.0001), emotional symptoms (*B* = 1.12, 95%CI 0.87–1.36, *p* < 0.0001), and conduct problems (*B* = 0.66, 95%CI 0.44–0.88, *p* < 0.0001), when adjusting for variables relating to the child and their family environment (Table 3). Evidence for an association remained in Model 2 after adjustment for the other SDQ subscales (hyperactivity symptoms *B* = 0.25, 95%CI 0.07–0.43, *p* = 0.006; emotional symptoms *B* = 0.91, 95%CI 0.67–1.14, *p* < 0.0001; conduct problems *B* = 0.25, 95%CI 0.05–0.46, *p* = 0.013).

**TABLE 3:** Analysis 2: B coefficients for child emotional and behavioural difficulties (as measured by Strengths and Difficulties Questionnaire (SDQ)) according to presence of intellectual or developmental disability (IDD) with or without co‐occurring autism spectrum disorders (ASD) using hierarchical multiple regression (*n* = 1904)

SDQ Child emotional and behavioural difficulties	Model 1		Model 2	
	B (95% CI)	*p*‐value	B (95% CI)	*p*‐value
SDQ total score	3.7 (3.18, 4.30)	<0.0001	‐‐	‐‐
Hyperactivity symptoms	0.51 (0.32, 0.70)	<0.0001	0.25 (0.07, 0.43)	0.006
Emotional symptoms	1.12 (0.87, 1.36)	<0.0001	0.91 (0.67, 1.14)	<0.0001
Conduct problems	0.66 (0.44, 0.88)	<0.0001	0.25 (0.05, 0.46)	0.013

*Note*: Hierarchical multiple regression. **Model 1** –associations between IDD with or without ASD and mental health diagnosis including confounding variables – child sex, child developmental level, child physical disability, deprivation. **Model 2** –Model 1 including other SDQ subscales.

Abbreviation: CI, confidence interval.

### Analysis 3: The association between intellectual or developmental disability with and without co‐occurring autism spectrum disorders and parental psychological distress as indexed by the Everyday Feelings Questionnaire

A hierarchical multiple regression model was conducted to investigate the associations between IDD with and without co‐occurring ASD, and parental psychological distress. Model 1 adjusted for deprivation, developmental level, physical disability, and Model 2 for the SDQ mental health symptoms (hyperactivity, emotional problems and conduct problems subscales).

In Model 1 there was evidence that having ASD in addition to IDD significantly predicted parental psychological distress (*β* = 0.1, 95% CI 0.85 to 2.21, *p* < 0.0001, Table 4). The evidence for this association remained significant in Model 2 after adjustment for other SDQ mental health difficulty symptom scales (*β* = 0.05, 95% CI 0.05–1.40, *p* = 0.04, Table 4). Mental health symptoms of hyperactivity (*β* = 0.13, 95% CI 0.29–0.63, *p* < 0.0001), emotional difficulties (*β* = 0.15, 95% CI 0.26–0.51, *p* < 0.0001) and conduct difficulties (*β* = 0.07, 95% CI 0.07–0.37, *p* < 0.004) all significantly contributed to the model.

**TABLE 4:** Analysis 3: B coefficients for parental psychological distress (Everyday Feelings Questionnaire (EFQ)) according to presence of mental health symptoms (Strengths and Difficulties Questionnaire (SDQ) subscale scores) using hierarchical multiple regression (*n* = 1904)

Parent psychological distress	B (95% CI)	Standardised *β*	*p*‐value
**Model 1**			
ASD (0 no ASD/1 ASD)	1.53 (0.85, 2.21)	0.10	<0.0001
**Model 2**			
ASD (0 no ASD/1 ASD)	0.73 (0.05, 1.40)	0.05	0.04
SDQ hyperactivity symptoms	0.46 (0.29, 0.63)	0.13	<0.0001
SDQ emotional symptoms	0.38 (0.26, 0.51)	0.15	<0.0001
SDQ conduct symptoms	0.22 (0.07, 0.37)	0.07	0.004

*Note*: **Model 1** –associations between IDD with or without ASD including confounding variables—child sex, developmental level, physical health and deprivation. **Model 2** –Model 1 including SDQ subscales as covariates.

Abbreviation: CI, confidence interval.

### Sensitivity analyses

All analyses were repeated with the subgroup of participants who completed assessments online without assistance form the study team. No differences in the patterns of significance were found. Results are presented in the Supplementary materials (Tables S2‐S4).

## DISCUSSION

Previous studies on relatively small samples have suggested that children with both IDD and ASD are at increased risk of associated mental health and neurodevelopmental symptoms (hyperactivity, emotional difficulties, conduct problems) compared to those with IDD alone on measures such as the SDQ (Totsika, Hastings, Emerson, Berridge, et al., 2011; Totsika, Hastings, Emerson, Lancaster, et al., 2011). This is the first study to evaluate whether those findings could be replicated in a cohort of children with IDD of genetic aetiology. The replication study, based on a large national cohort, found that the presence of ASD in addition to IDD put children at twice the risk of developing emotional disorders and disruptive behaviour disorders, as well as neurodevelopmental conditions such as ADHD. Parents of children with IDD and co‐occurring ASD were also reported higher levels of psychological distress than those with IDD alone. Our findings suggest that the additional mental health and behavioural symptoms in those with ASD contributed to the degree of parental psychological distress.

Anxiety disorders are one of the most common co‐occurring conditions in children with ASD without IDD (Lai et al., 2019), but they are also frequent in children with IDD (Emerson et al., 2007; Simonoff et al., 2008). In our supplementary analyses GAD was more common in IMAGINE children who met criteria for ASD than IDD alone. There also appeared to be no difference in the likelihood of having separation anxiety, social phobia or specific phobia in those with or without ASD. However, as few IMAGINE children met criteria for these disorders (*n* < 49), it is likely that these null findings cannot be interpreted, as the analyses were under‐powered.

Depression was infrequently diagnosed in the cohort, but most were not in a high‐risk age group (median age 9 years). Depression often goes undetected in young people with IDD and/or ASD, because such internalising symptoms are not easy to detect by observation (McBrien, 2003).

IMAGINE cohort children often had symptoms of ODD, but not CD, which is compatible with the finding that among children with ASD in general, ODD is not uncommon (28%, Simonoff et al., 2008). ODD is rather less frequently found in those with IDD alone (11%; Emerson et al., 2007). At this stage in our analyses we have not quantified differences in the presentation of ODD symptoms (angry and irritable symptoms, argumentative and defiant behaviour, and vindictiveness) in the two groups but should such differences exist, they could inform the choices of appropriate behaviour management.

In neurotypical and autistic populations, co‐occurring physical health problems, sex and socio‐economic deprivation, individually influence the nature of mental health difficulties (Boyd et al., 2015; Emerson & Hatton, 2007; Emerson et al., 2010; Kuehner, 2017; Lai et al., 2019; Reiss, 2013; Sedgewick et al., 2021; Seedat et al., 2009; Shahtahmasebi et al., 2011; Tsakanikos et al., 2011; Visser et al., 2021). We adjusted for these factors in our analyses and found that the increase in DSM‐5 diagnoses of those with co‐occurring IDD and ASD was not fully accounted for by either socio‐economic deprivation, sex, developmental level or physical health problems. However, there may be other individual and environmental factors, which were not included in this analysis, that account for the increased likelihood of additional diagnoses in those children with autism.

Parents of children with both IDD and ASD, reported higher levels of parental distress. Previous studies of the relationship between mental health problems and parental psychological distress in those with ASD, IDD or both disorders have found inconsistent results (Blacher & McIntyre, 2006; Eisenhower et al., 2005; Griffith et al., 2010; Herring et al., 2006; Totsika, Hastings, Emerson, Berridge, et al., 2011). Some studies have concluded that a child's additional mental health difficulties account for the increase in parental distress (Blacher & McIntyre, 2006; Herring et al., 2006), while others have found that the parental psychological distress remains high, even after adjusting for their child's additional mental health difficulties (Eisenhower et al., 2005; Griffith et al., 2010; Totsika, Hastings, Emerson, Berridge, et al., 2011).

Measured dimensionally, emotional, hyperactivity and conduct symptoms all significantly contributed to parental psychological distress in those with ASD. Comparing standardised beta coefficients, emotional symptoms (0.15) had the strongest impact on parental psychological distress, followed by hyperactivity (0.13) and conduct symptoms (0.07). It is possible the characteristic behavioural features of ASD (e.g., social difficulties, cognitive rigidity) also contributed to parental psychological distress. Interestingly, although ADHD and disruptive behaviour disorders are more than twice as prevalent compared to emotional disorders in those with co‐occurring IDD and ASD, the child's emotional symptoms were more strongly associated with parental EFQ scores in this cohort.

The finding that the presence of additional mental health symptoms (emotional, hyperactivity, conduct) is associated with greater parental psychological distress could also be explained by a shared genetic background. It is possible that both the parent and the child share genetic factors that are associated with an increased risk of mental health difficulties (Grotzinger, 2021; Martin et al., 2017). We have not controlled for parent genetic profile or family history of mental health difficulties in this analysis, but acknowledge that this may be a contributory factor. Another possibility is that there is a bidirectional relationship between child and parental mental health (Zaidman‐Zait et al., 2014), which we have not accounted for as part of this study. Further research is needed to understand the influence of the genetic and environmental factors contribute to parent and child mental health outcomes throughout development.

Our study needs to be interpreted in light of several limitations. Firstly, to take part in the study, participants were required to have a known genetic diagnosis and we do not have data on potentially asymptomatic individuals with similar genomic disorders; therefore, our findings are not applicable to the general population. Our study concerns those who are ascertained as having IDD of genetic cause and our findings are likely to hold true in this population. Another limitation of this study is that participants were not assessed in person and assessments were completed by online parent report. The continuous and categorical measures of mental health and neurodevelopment were completed by the same informant, using an online methodology that is well‐established in the English national mental health surveys of children and young people and allows data collection on a large cohort as well as a standardised assessment of mental health (Green et al., 2004). However, only a small proportion of participants in those surveys had IDD. The DAWBA assessment procedures were subjected to rigorous validation to ensure they were comparable to the UK national cohort study diagnostic rating procedures (Wolstencroft et al., 2021) and, the ASD DAWBA module was further validated against clinical ratings on the 3Di‐sv in a sub‐sample of IMAGINE participants with excellent sensitivity and specificity. In addition, it must be noted that the current data is cross‐sectional and that the sample comprises children of different age groups and developmental abilities, further work examining the longitudinal nature of co‐occurring mental health difficulties, such as when they arise may be of benefit regarding the most beneficial timing for intervention. The IMAGINE 2 study aims to address these questions by following up the cohort longitudinally. Although we adjusted for demographic data and IMD, we did not include other factors of potential relevance therefore, residual and/or unmeasured confounding remains possible. Genetic confounding which we did not fully account for, must also be considered, particularly with regard to parental mental health analyses. Although this study is the largest of its kind, it is based only on the UK population, and we hope that it will generate future research including cross‐cultural comparisons.

Large scale studies, such as IMAGINE, allow us to chart the needs of children with IDD with rare genetic disorders and plan service delivery around a genetics‐first healthcare approach. The clinical implications of our findings are to highlight the importance of a comprehensive mental health and neurodevelopmental assessment of children with ASD and IDD, and a holistic approach to treatment, which considers all of the child's presenting concerns, rather than addressing them in isolation. The diagnosis of a pathogenic variant in children with IDD should be taken as a predictor of possible child mental health problems. The identification of co‐occurring ASD should prompt a comprehensive neurodevelopmental and mental health assessment. Early psycho‐social support is needed to prevent poor mental health outcomes in later childhood. At present this need is not being met; less than a third (28.9%) of families in IMAGINE reported receiving help from a mental health professional. In addition, in those of IDD of genetic cause we need to understand more about specific genomic disorders, so that we can understand whether some risks are related to shared underlying pathways and others related to levels of support, so that we can leverage the information offered by genetic testing to tailor support and advice to children and their families. The level of parental psychological distress in IDD cohort was high, and higher still in those with co‐occurring ASD.

The efficacy of interventions will be enhanced by identifying other factors such as professional and educational support, family support, or sleep quality etc., which may influence parental psychological distress. Understanding the transactional relationship between parental psychological distress and child behavioural difficulties in children with IDD with and without ASD will help identify the most effective timing for psycho‐social intervention.

## CONCLUSION

Rapid advances in genetic testing technology mean that the National Health Service (NHS) is identifying more children with IDD of genetic aetiology at a young age (<4 years). The IMAGINE consortium has shown that these children are at a high risk of developing mental health conditions and neurodevelopmental disorders. Knowing the strength of association between a specified genetic diagnosis and later risk of neurodevelopmental and mental health problems presents a unique opportunity to offer tailored early intervention and psycho‐social support to families. Our findings show that the prevalence rates of mental health and neurodevelopmental disorder are elevated in those with a co‐occurring ASD relative to those IDD alone. The risk of co‐occurring mental health difficulties was nearly doubled for ADHD, emotional disorders and disruptive behaviour disorders in children with both IDD and ASD. Parents raising IDD children with co‐occurring ASD also reported higher levels of psychological distress compared to those with IDD alone. The presence of ASD in children with IDD of genetic aetiology should be known to both paediatric and mental health teams. Their increased risk of behavioural and emotional problems indicates the need for careful monitoring because they and their parents may require additional behavioural and mental health support.

## AUTHOR CONTRIBUTIONS

**Jeanne Wolstencroft**: Conceptualization; Data curation; Formal analysis; Investigation; Methodology; Project administration; Writing – original draft; Writing – review & editing. **Ramya Srinivasan**: Formal analysis; Writing – original draft; Writing – review & editing. **Jeremy Hall**: Conceptualization; Funding acquisition; Investigation; Methodology; Writing – review & editing. **Marianne B. M. van den Bree**: Conceptualization; Funding acquisition; Investigation; Methodology; Writing – review & editing. **Michael J. Owen**: Conceptualization; Funding acquisition; Investigation; Methodology; Writing – review & editing. **IMAGINE Consortium**: Conceptualization; Data curation; Resources. **F. Lucy Raymond**: Conceptualization; Data curation; Funding acquisition; Investigation; Methodology; Writing – review & editing. **David Skuse**: Conceptualization; Funding acquisition; Investigation; Methodology; Project administration; Supervision; Writing – original draft; Writing – review & editing.

## CONFLICT OF INTEREST

MJO, MvdB and JH have a research grant from Takeda Pharmaceuticals that is outside the scope of the present study. The remaining authors have declared that they have no competing or potential conflicts of interest.

## ETHICAL CONSIDERATIONS

All procedures were approved by London–Queen Square Research Ethics Committee (14/LO/1069).

## Supporting information

Supplementary Information S1

## Data Availability

The full phenotypic IMAGINE dataset is available from the UK Data Archive under special license access (SN 8621): https://beta.ukdataservice.ac.uk/datacatalogue/studies/study?id=8621. Requests for genotype or linked genotypic‐phenotypic data can be made through the study's data access committee: https://imagine‐id.org/healthcare‐professionals/datasharing/.

## APPENDIX 1 SuppIMAGINE‐ID consortium members.

### IMAGINE‐ID Consortium author list

**Table:**

Surname	Initials	First Name	Title	Institution
Raymond	F L	F Lucy	Professor	Department of Medical Genetics, University of Cambridge, UK
Dewhurst	E	Eleanor	Mrs	Department of Medical Genetics, University of Cambridge, UK
Lafont	A	Amy	Mrs	Department of Medical Genetics, University of Cambridge, UK
Timur	H	Husne	Ms	Department of Medical Genetics, University of Cambridge, UK
Wicks	F	Francesca	Mrs	Department of Medical Genetics, University of Cambridge, UK Unique Charity, UK
Ye	Z	Zheng	Dr	Department of Medical Genetics, University of Cambridge, UK
Baker	K	Kate	Dr	Department of Medical Genetics, University of Cambridge, UK
Walker	N	Neil	Dr	Department of Medical Genetics, University of Cambridge, UK
Wallwork	S	Sarah	Ms	Department of Medical Genetics, University of Cambridge, UK
Skuse	D	David	Professor	Great Ormond Street Institute of Child Health, University College London, UK
Denaxas	S	Spiros	Dr	Institute of Health Informatics, University College London, London, UK
Mandy	W	William	Dr	Division of Psychology & Language Sciences, University College London, UK
Wolstencroft	J	Jeanne	Dr	Great Ormond Street Institute of Child Health, University College London, UK
Davies	S	Sarah	Ms	Great Ormond Street Institute of Child Health, University College London, UK
Erwood	M	Marie	Ms	Great Ormond Street Institute of Child Health, University College London, UK
Juj	M	Manoj	Mr	Great Ormond Street Institute of Child Health, University College London, UK
Kerry	E	Eleanor	Ms	Great Ormond Street Institute of Child Health, University College London, UK
Lucock	A	Anna	Ms	Great Ormond Street Institute of Child Health, University College London, UK
Printzlau	F	Frida	Ms	Great Ormond Street Institute of Child Health, University College London, UK
Srinivasan	R	Ramya	Dr	Great Ormond Street Institute of Child Health, University College London, UK
Walker	S	Susan	Dr	Great Ormond Street Institute of Child Health, University College London, UK
Coscini	N	Nadia	Dr	Great Ormond Street Institute of Child Health, University College London, UK
Fatih	N	Nasrtullah	Mr	Great Ormond Street Institute of Child Health, University College London, UK
Denyer	H	Hayley	Ms	Great Ormond Street Institute of Child Health, University College London, UK
Andrews	S	Sophie	Ms	MRC Centre for Neuropsychiatric Genetics and Genomics, Division of Psychological Medicine and Clinical Neurosciences, Cardiff University, UK
Chawner	SJRA	Samuel	Dr	MRC Centre for Neuropsychiatric Genetics and Genomics, Division of Psychological Medicine and Clinical Neurosciences, Cardiff University, UK
Cuthbert	A	Andrew	Dr	MRC Centre for Neuropsychiatric Genetics and Genomics, Division of Psychological Medicine and Clinical Neurosciences, Cardiff University, UK
Challenger	A	Aimee	Ms	MRC Centre for Neuropsychiatric Genetics and Genomics, Division of Psychological Medicine and Clinical Neurosciences, Cardiff University, UK
Hall	J	Jeremy	Professor	MRC Centre for Neuropsychiatric Genetics and Genomics, Division of Psychological Medicine and Clinical Neurosciences, Cardiff University, UK
Lewis	N	Nicola	Ms	MRC Centre for Neuropsychiatric Genetics and Genomics, Division of Psychological Medicine and Clinical Neurosciences, Cardiff University, UK
Owen	MJ	Michael	Professor Sir	MRC Centre for Neuropsychiatric Genetics and Genomics, Division of Psychological Medicine and Clinical Neurosciences, Cardiff University, UK
Ray	S	Sinead	Ms	MRC Centre for Neuropsychiatric Genetics and Genomics, Division of Psychological Medicine and Clinical Neurosciences, Cardiff University, UK
Sopp	M	Matthew	Mr	MRC Centre for Neuropsychiatric Genetics and Genomics, Division of Psychological Medicine and Clinical Neurosciences, Cardiff University, UK
Moss	H	Hayley	Ms	MRC Centre for Neuropsychiatric Genetics and Genomics, Division of Psychological Medicine and Clinical Neurosciences, Cardiff University, UK
van den Bree	MBM	Marianne	Professor	MRC Centre for Neuropsychiatric Genetics and Genomics, Division of Psychological Medicine and Clinical Neurosciences, Cardiff University, UK
Holmans	P	Peter	Professor	MRC Centre for Neuropsychiatric Genetics and Genomics, Division of Psychological Medicine and Clinical Neurosciences, Cardiff University, UK
Bowen	S	Samantha	Ms	MRC Centre for Neuropsychiatric Genetics and Genomics, Division of Psychological Medicine and Clinical Neurosciences, Cardiff University, UK
Bradley	K	Karen	Mrs	MRC Centre for Neuropsychiatric Genetics and Genomics, Division of Psychological Medicine and Clinical Neurosciences, Cardiff University, UK
Birch	B	Philippa	Ms	MRC Centre for Neuropsychiatric Genetics and Genomics, Division of Psychological Medicine and Clinical Neurosciences, Cardiff University, UK
Tong	M	Molly	Ms	MRC Centre for Neuropsychiatric Genetics and Genomics, Division of Psychological Medicine and Clinical Neurosciences, Cardiff University, UK
Ford	T	Tasmin	Professor	Department of Psychiatry, University of Cambridge
Wynn	S	Sarah	Ms	Unique Charity, UK
Robertson	L	Lisa	Dr	Aberdeen Royal Infirmary Genetics Service
Berg	J	Jonathan	Dr	Ninewells Hospital Dundee Genetics Service
Lampe	A	Anne	Professor	Western General Hospital Edinburgh Genetics Service
Joss	S	Shelagh	Dr	Glasgow Genetics Centre, Glasgow
Brennan	P	Paul	Dr	Northern Genetics Service, Newcastle
Kraus	A	Alison	Dr	Yorkshire Regional Genetics Service ‐ Clinical Genetics
Weber	A	Astrid	Dr	Cheshire & Merseyside Regional Genetic Service
Rawson	M	Myfanwy	Ms	Manchester Centre for Genomic Medicine
Johnson	D	Diana	Dr	Sheffield Genetic Services
Vasudevan	P	Pradeep	Dr	Leicestershire Genetics Centre, Leicester
Harrison	R	Rachel	Dr	Nottingham Regional Genetics Service
Williams	D	Denise	Dr	West Midlands Regional Genetics Service, Birmingham
Maher	E	Eamonn	Professor	East Anglian Medical Genetics Service, Cambridge
Kini	U	Usha	Dr	Oxford Genetics Service
Clowes	V	Virginia	Dr	London North West Thames Regional Genetics Service
Gurasashvili	J	Jana	Ms	London North Thames Genomic Laboratory Hub, Great Ormond Street Hospital, London
Mansour	S	Sahar	Dr	London South West Thames Regional Genetics Service, St Georges Hospital, Tooting, London
Holder‐Espinasse	M	Muriel	Dr	London South East Thames Regional Genetics Service Guy's Hospital, London
Watford	A	Amy	Ms	Bristol Clinical Genetics Service, Bristol
Rankin	J	Julia	Dr	Peninsula Genetics Service, Exeter
Baralle	D	Diana	Dr	Wessex Clinical Genetics Service
Procter	A	Annie	Dr	All Wales Regional Genetics Service
Fleur van Dijk	F	Fleur	Dr	London North West Thames Regional Genetics Service
