# Supplementary material for: Mental health impact of autism on families of children with intellectual and developmental disabilities of genetic origin
Source: JCPP Adv. 2023 Jan 13;3(1):e12128. doi: 10.1002/jcv2.12128 (PMC10241472; doi:10.1002/jcv2.12128)
Supplement: Supplementary file 1 — Supplementary Information S1 [file JCV2-3-e12128-s001.docx]

**Supporting Information**

**Data Accessibility**

The full phenotypic IMAGINE dataset is available from the UK Data Archive under special license access (SN 8621): <https://beta.ukdataservice.ac.uk/datacatalogue/studies/study?id=8621>

Requests for genotype or linked genotypic-phenotypic data can be made through the study’s data access committee: <https://imagine-id.org/healthcare-professionals/datasharing/>. Please see an example of the genetic data available in the eTable1 below.

**Table S1 – Analysis 1: Odds ratios for child mental health diagnosis according to presence of IDD with or without co-occurring ASD using binary logistic regression models (n=1904)**

| **DAWBA Child DSM-V diagnosis**  (0 no ASD/ 1 ASD) | **Model 1** | | **Model 2** | | **Model 3** | |
| --- | --- | --- | --- | --- | --- | --- |
|  | OR (95% CI) | p-value | OR (95% CI) | p-value | OR (95% CI) | p-value |
| Generalised anxiety | 2.44  (1.69, 3.50) | **<0.001** | 2.30  (1.58, 3.34) | **<0.001** | 2.15  (1.46, 3.16) | **<0.001** |
| Specific Phobia | 1.54  (0.87, 2.71) | 0.14 | 1.5  (0.84, 2.68) | 0.17 | 1.34  (0.74, 2.4) | 0.34 |
| Social Phobia | 0.86  (0.8, 9.48) | 0.9 | 0.60  (0.05, 7.53) | 0.69 | 0.57  (0.02, 15.59) | 0.70 |
| Separation Anxiety | 1.15  (0.58, 2.27) | 0.69 | 1.08  (0.54, 2.16) | 0.84 | 0.81  (0.39, 1.66) | 0.56 |
| OCD | 1.72  (0.11, 27,50) | 0.70 | 2.29  (0.14, 37.68) | 0.56 | 1.69  (0.09, 32.65) | 0.73 |
| Depression | 2.87  (0.69, 12.06) | 0.15 | 2.39  (0.55, 10.45) | 0.25 | 2.15  (0.37, 12.40) | 0.39 |
| ODD | 2.39  (1.82, 3.13) | **<.001** | 2.25  (1.71, 2.96) | **<.001** | 1.9  (1.44, 2.56) | **<0.001** |
| CD | 1.73  (0.77, 3.87) | 0.18 | 1.48  (0.66, 3.36) | 0.34 | 1.07  (0.46, 2.50) | 0.87 |

**Model 1** – univariable associations between IDD with or without co-occurring ASD and DSM-5 diagnosis

**Model 2** – Model 1 including confounding variables – child sex, child developmental level, deprivation (IMD), child physical disability

**Model 3** – Model 2 including additional co-occurring DSM-5 diagnoses as confounding variables.

**Table S2 – Analysis 1: Odds ratios for child mental health diagnosis according to presence of IDD with or without co-occurring ASD using binary logistic regression models (n=1637)**

| **DAWBA Child DSM-5 diagnosis**  (0 no ASD/ 1 ASD) | **Model 1** | | **Model 2** | | **Model 3** | |
| --- | --- | --- | --- | --- | --- | --- |
|  | OR (95% CI) | p-value | OR (95% CI) | p-value | OR (95% CI) | p-value |
| Attention Deficit Hyperactivity disorder | 2.13  (1.68, 2.71) | <0.0001 | 2.03  (1.59, 2.59) | <0.0001 | 1.80  (1.39, 2.32) | <0.0001 |
| Emotional disorders | 2.17  (1.56, 3.03) | <0.0001 | 2.13  (1.51, 3.00) | <0.0001 | 1.97  (1.40, 2.79) | <0.0001 |
| Generalised anxiety | 2.47  (1.66, 3.69) | <0.0001 | 2.35  (1.56, 3.54) | <0.0001 | 2.18  (1.43, 3.33) | 0.0003 |
| Specific Phobia | 1.65  (0.87, 3.15) | 0.13 | 1.67  (0.87, 3.22) | 0.13 | 1.46  (0.74, 2.89) | 0.27 |
| Separation Anxiety | 1.02  (0.45, 2.32) | 0.96 | 0.93  (0.40, 2.16) | 0.86 | 0.67  (0.28, 1.62) | 0.37 |
| Disruptive behaviour disorders | 2.30  (1.72, 3.07) | <0.0001 | 2.16  (1.61, 2.90) | <0.0001 | 1.79  (1.31, 2.43) | 0.0002 |
| Oppositional Defiant Disorder | 2.38  (1.77, 3.2) | <0.0001 | 2.24  (1.65, 3.03) | <0.0001 | 1.9  (1.39, 2.63) | <0.0001 |
| Conduct Disorder | 1.67  (0.73, 3.82) | 0.22 | 1.40  (0.60, 3.24) | 0.43 | 0.97  (0.41, 2.33) | 0.95 |

**Model 1** – univariable associations between IDD with or without co-occurring ASD and DSM-5 diagnosis

**Model 2** – Model 1 including confounding variables – child sex, child developmental level, deprivation (IMD), child physical disability

**Model 3** – Model 2 including co-occurring DSM-5 diagnoses as confounding variables.

Model results for social phobia, OCD and depression are not presented as ASD cases n<5.

**Table S3 – Analysis 2: B coefficients for child emotional and behavioural difficulties (as measured by SDQ) according to presence of IDD with or without co-occurring ASD using hierarchical multiple regressions (n=1637)**

| **SDQ Child emotional and behavioural difficulties** | **Model 1** | | **Model 2** | |
| --- | --- | --- | --- | --- |
|  | B (95% CI) | p-value | B (95% CI) | p-value |
| SDQ total score | 3.71  (3.10, 4.33) | <0.0001 | -- | -- |
| Hyperactivity symptoms | 0.48  (0.28, 0.69) | <0.0001 | 0.23  (0.04, 0.43) | 0.02 |
| Emotional symptoms | 1.10  (0.84, 1.37) | <0.0001 | 0.89  (0.64, 1.15) | <0.0001 |
| Conduct problems | 0.64  (0.40, 0.88) | <0.0001 | 0.24  (0.02, 0.46) | 0.03 |

**Model 1** –associations between IDD with or without ASD and mental health diagnosis including confounding variables – child sex, child developmental level, child physical disability, deprivation

**Model 2** –Model 1 including other SDQ subscales

**Table S4 – Analysis 3: B coefficients for parental psychological distress (EFQ) according to presence of mental health symptoms (SDQ subscale scores) using hierarchical multiple regression (n=1637)**

| **Parent psychological distress** | **B (95% CI)** | **Standardised β** | **p-value** |
| --- | --- | --- | --- |
| **Model 1** | | | |
| ASD (0 no ASD/ 1 ASD) | 1.74 (1.01, 2.47) | 0.12 | <0.0001 |
| **Model 2** | | | |
| ASD (0 no ASD/ 1 ASD) | 0.95 (0.23, 1.67) | 0.06 | 0.01 |
| SDQ Hyperactivity symptoms | 0.45 (0.27, 0.63) | 0.13 | <0.0001 |
| SDQ Emotional Symptoms | 0.38 (0.24, 0.51) | 0.15 | <0.0001 |
| SDQ Conduct symptoms | 0.25 (0.09, 0.41) | 0.08 | 0.003 |

**Model 1** –associations between IDD with or without ASD including confounding variables – child sex, developmental level, physical health and deprivation

**Model 2** –Model 1 including SDQ subscales as covariates
